# Supplementary material for: Effects of the Early Phase of COVID-19 on the Autistic Community in Sweden: A Qualitative Multi-Informant Study Linking to ICF
Source: Int J Environ Res Public Health. 2022 Jan 24;19(3):1268. doi: 10.3390/ijerph19031268 (PMC8835079; doi:10.3390/ijerph19031268)
Supplement: Supplementary file 1 [file ijerph-19-01268-s001.zip › ijerph-1514959-Supplementary.pdf]

Supplemental Material

The Swedish response to COVID-19

General information On the 1st of February 2020, the Swedish Government classified COVID-19 as a disease that constitutes a danger to society. The decision opened up the possibility to implement extraordinary disease control measures. While the Swedish government will clarify Swedish laws in regulations ("förordning"), expert agencies including the Public Health Agency is tasked with providing even more detailed rules and advise ("föreskrifter"; "rekommendationer och allmänna råd") according to their expertise, i.e. disease control for individuals in Sweden. In addition to this, the Public Health Agency is tasked with dissemination of evidence-based and expert knowledge. While Swedish officials have reviewed the situation under COVID-19 constantly and implemented regulations and rules accordingly, the general aim of the measures taken by the government and the governmental agencies (including the Public Health Agency) has been to reduce the spread of the virus (to "flatten the curve") so that large numbers do not become ill at the same time. It is communicated that effects of the spread of the virus has been weighed against the effects on society and public health in general.

Development of spread of COVID-19 The strategy for testing COVID-19 in the population has shifted over time. Initially, testing was restricted to certain groups; individuals who travelled to affected foreign areas, which needed inpatient care and healthcare providers (hospital, elderly care and institutional care; though regional differences in tracing). In may 2020 the government tasked the Public Health Agency to develop a strategy for scaling up testing and from the 5th of June 2020 the Public Health Agency has successively been able to scale up testing (laboratory analyses, sample collection methods etc.) in Swedish regions while prioritising patients in in-patent care and healthcare providers. There are regional differences in the spread of the virus where some areas, e.g. Stockholm region, has been particularly affected.

Following these premises, around at the height of the spread in week 15 of 2020 there were 10800 confirmed cases (with 3696 reported week 15), 1098 registered deaths (87% of which were 70 years or older) and average 39 people admitted to intensive care units/day (Public Health Agency: report w week 15). At the time of the study-interviews, Sweden had 79126 confirmed cases (with 1376 reported week 30; 87% reported mild symptoms), 5712 registered deaths (89% of which were 70 years or older) and 8 admitted to intensive care units over the whole week (Public Health Agency: report week 30).

Regulations imposed by the government

In order to adhere with reports from expertise agencies, including recommendations and rules presented by the Public Health Agency, the Swedish government has presented several regulations from March onwards. Regulations have included extended benefits from the Social Insurance Agency in case of illness, increased access to unemployment benefits funds, and short-time work allowance. In addition, public gatherings were restricted to 500 individuals and, by the end of March lowered to a maximum of 50 individuals. The Foreign Ministry regulated all non-essential travel to foreign countries from March onwards, with some exceptions depending on changing international circumstances.

Rules presented by the Public Health Agency

Rules to further specify laws and regulations have been published by the Public Health Agency starting from March 2020. The responsibility to implement these rules falls on individuals and, in extension working businesses, according to The Communicable Diseases Act. Other actors may assist; for instance, the Police Authority may disperse public gatherings.

Rules include general hygienic advice, remaining at home in case of symptoms, keeping appropriate physical distancing (e.g. avoiding public transport as well as group gatherings and activities whenever possible), and proper behaviours when suspected or confirmed diagnosis of Covid-19. Specific rules have been clarified for comportment of places of work (e.g. working from home whenever possible and/or allowing workings to comply with general rules at their places of work), shopping, sports activities and organisation to name a few. From the 24th of March specific rules where published for service businesses, including bars and restaurants which would allow implementation of the general rules. The Public Health Agency has specifically addressed several public holidays to allow individuals to comply with general rules.

From the 17th of March it was recommended that high schools and universities implemented remote schooling in order to restrict travel and gatherings as well as to protect the elderly. It was communicated that younger children and primary school children were not major contributors to the spread of the virus wherefore pre- and primary schools have remained open throughout. However, following rules to stay at home when showing the slightest symptoms, younger children may have, in effect, been learning from home over time periods as well.

From the 16th of March the Public Health Agency recommended that individuals over the age of 70 restrict any social-physical contacts, following their specific vulnerability to COVID-19. Specific guidelines for implementing hygiene-rules by elderly care takers have been stressed and published by expert agencies, including the Public Health Agency, and training opportunities has been offered. Since March the Public Health Agency and the government jointly recommended that visitors were not welcome at elderly care homes, since the April 1st there has been a national prohibition to visit elderly care homes at all.

Effects of measures

The Swedish strategy has raised some controversy and the efficiency of measures have been debated, a discussion which is outside the scope of this general overview. The Public Health Agency has cooperated with a major network operator to investigate effects on travel within Sweden, starting in April 2020 (Telia). Reports suggest marked decreases 12-19th of March, dates that co-occur with recommendation to distance learning and working from home. From the end of March travelling stabilised. There was an increase in travel from May onwards, which was comparable to travelling patterns in February 2020.
